# Underage Youth and Young Adult e-Cigarette Use and Access Before and During the Coronavirus Disease 2019 Pandemic

**DOI:** 10.1001/jamanetworkopen.2020.27572

**Published:** 2020-12-03

**Authors:** Shivani Mathur Gaiha, Lauren Kass Lempert, Bonnie Halpern-Felsher

**Affiliations:** 1 Division of Adolescent Medicine, Department of Pediatrics, Stanford University, Palo Alto, California; 2 Center for Tobacco Control Research & Education, University of California, San Francisco

## Abstract

**Importance:**

Understanding patterns of e-cigarette use and access during the coronavirus disease 2019 (COVID-19) pandemic is important because e-cigarettes may put users at risk for more severe respiratory effects and other health problems.

**Objective:**

To examine whether underage youth and young adults who ever used e-cigarettes self-reported changes in access and use of e-cigarettes since the COVID-19 pandemic began.

**Design, Setting, and Participants:**

A national, cross-sectional online survey study was conducted from May 6 to May 14, 2020. This sample of 4351 participants aged 13 to 24 years across the US included 2167 e-cigarette ever-users. Quota sampling was used to balance for age, sex, race/ethnicity, and 50% having ever used e-cigarettes.

**Main Outcomes and Measures:**

Change in e-cigarette use (increase, decrease, quit, no change, and switch to another product) and access to e-cigarettes (easier or harder, and change in point-of-purchase) before and after the COVID-19 pandemic began, reasons for change, number of times e-cigarettes were used, nicotine dependence, and sociodemographic data.

**Results:**

This study focused on 2167 e-cigarette ever-users among 4351 participants who completed the survey. Among 2167 e-cigarette users, a total of 1442 were younger than 21 years and 725 were aged 21 years or older; 1397 were female (64.5%) and 438 identified as lesbian, gay, bisexual, transgender, queer (20.2%). The survey completion rate was 40%. Since the COVID-19 pandemic began, 1198 of 2125 e-cigarette users (56.4%) changed their use: 388 individuals (32.4%) quit, 422 individuals (35.3%) reduced the amount of nicotine, 211 individuals (17.6%) increased nicotine use, 94 individuals (7.8%) increased cannabis use, and 82 individuals (6.9%) switched to other products. Participants reported that not being able to go to vape shops and product unavailability were the reasons accessing e-cigarettes was difficult after the pandemic began. Since the COVID-19 pandemic began, individuals reported purchasing from alternative retail stores (disposables, 150 of 632 [23.7%]; pod-based, 144 of 797 [18.1%]; and other e-cigarette, 125 of 560 [22.3%], ie, between 18.1% and 23.7%), purchasing online instead of retail (disposables, 115 of 632 [18.2%]; pod-based, 156 of 797 [19.6%]; and other e-cigarette, 111 of 560 [19.8%], ie, between 18.2% to 19.8%), and shifted to retail instead of online (disposables, 11 of 632 [1.7%]; pod-based, 17 of 797 [2.0%]; and other e-cigarette, 13 of 560 [2.3%], ie, between 1.7%-2.3%). Other individuals reported no change: from retail stores (disposables 262 of 632 [41.5%]; pod-based 344 of 797 [43.2%]; and other e-cigarette, 223 of 560 [39.8%], ie, between 39.8% and 43.2%) and online (disposables 94 of 632 [14.9%]; pod-based 136 of 797 [17.1%]; and other e-cigarette, 88 of 560 [15.8%], ie, between 14.9% and 17.1%). Underage youth reported e-cigarette deliveries from vape shops and/or dealers or friends who received such deliveries, and 63 of 229 (27.5%) self-reported accessing e-cigarettes without age verification. e-Cigarette users were 52% less likely to quit or reduce their use if they previously used e-cigarettes between 11 and 99 times (adjusted odds ratio, 0.48; 95% CI, 0.30-0.78), 68% less likely to quit if they previously used e-cigarettes more than 100 times (adjusted odds ratio, 0.32; 95% CI, 0.20-0.51), and 51% were less likely to quit if they were nicotine dependent (adjusted odds ratio, 0.49; 95% CI, 0.35-0.70).

**Conclusions and Relevance:**

During the COVID-19 pandemic, youth e-cigarette users reported changes in e-cigarette use, point-of-purchase, and ability to purchase e-cigarettes without age verification. The US Food and Drug Administration and local policy makers may find these data useful to inform policies to prevent e-cigarette sales to underage youth.

## Key Points

**Question:**

Did underage youth and young adults (13-24 years) self-report changes in use and access to e-cigarettes during the coronavirus disease 2019 pandemic?

**Findings:**

In this national, online, cross-sectional survey study of 2167 youth and young adults using e-cigarettes, 1198 respondents reported changing their amount of e-cigarette use, with 810 reducing or quitting e-cigarette use; e-cigarette access shifted to alternative retail stores and online. Reduced e-cigarette use or quitting was associated with adhering to shelter-in-place guidelines and was less likely if participants had used e-cigarettes more than 10 times or were nicotine dependent.

**Meaning:**

Individuals younger than 21 years reported e-cigarette use and accessed e-cigarettes from online and retail stores during the coronavirus disease 2019 pandemic, suggesting a need to strengthen prevention of e-cigarette sales to such youth, including age verification, and provide cessation resources.

## Teaser

This survey study examines changes in use of e-cigarettes by individuals aged 24 years and younger during the coronavirus 2019 pandemic.

## Introduction

Despite federal legislation restricting the minimum age of sale of tobacco products to 21 years^1^ and guidance that e-cigarettes are unsafe for adolescent use,^2^ high rates of adolescent and young adult e-cigarette use have been reported. In 2019, 27.5% of high school students and 10.5% of middle school students reported past-month e-cigarette use.^3^ National data from 2018 indicated that 7.6% of young adults (18-24 years) reported past-month e-cigarette use.^4^ Widespread advertising and marketing,^5,6,7,8^ numerous flavors,^9,10,11^ concealability of products,^12,13^ curiosity,^10^and peer pressure^10^ are some reasons why underage youth use e-cigarettes. e-Cigarettes weaken the respiratory and immune system^14,15,16^ and tobacco and substance use are associated with users being more at risk of coronavirus disease 2019 (COVID-19).^17,18,19^ Thus, understanding patterns of e-cigarette use in light of COVID-19 is important.

Since early March 2020, most US states issued stay-at-home orders, mandating that individuals socially distance themselves from others to limit or prevent the spread of COVID-19. Before the pandemic, most adolescents reported that they obtained e-cigarettes from friends and brick-and-mortar retail stores, with fewer adolescents accessing e-cigarettes online.^20,21,22^ Since the number of COVID-19 infections began increasing in the US, many retail stores closed, including vape and smoke shops. Adolescents and young adults have been staying home with family, possibly leading to reduced socialization and reduced access to e-cigarettes from friends. The potential association of stay-at-home mandates and retail store closures with adolescents’ access to and use of e-cigarettes is unknown.

To our knowledge, there have been no population-based studies assessing and characterizing e-cigarette access and use among underage youth and young adults before and during the COVID-19 pandemic. Understanding such patterns and shifts may inform the development of timely and age-appropriate public health messaging and provide insights on policy levers for long-term prevention of underage access to and use of e-cigarettes. Using a national sample, we examined whether adolescents’ and young adults’ self-reported e-cigarette use changed, where they accessed e-cigarettes before and during the pandemic, reasons for change, and factors associated with dominant changes in use.

## Methods

### Participants

We used Qualtrics to recruit participants and field a national, cross-sectional, anonymous online survey among its existing US panel members. The survey was conducted from May 6 to May 14, 2020. Qualtrics meets ESOMAR standards for social and behavioral research and monitors and controls the quality of survey responses.^23^ Qualtrics provided its existing panelists with a link to a description of the survey (eg, via gaming sites, social media, customer loyalty portals, and website intercept recruitment). Panelists were informed that they could withdraw from the study at any time without any consequences. Panelists interested in completing our survey provided online consent and assent before beginning the self-administered survey, and panelists received financial compensation. Our sampling quota included 1:1:1 ratio of participants aged 13 to 17 years, 18 to 20 years, and 21 to 24 years, and a 50:50 ratio of ever-e-cigarette users to never-e-cigarette users. Our sample was further balanced on sex and race/ethnicity per US Census data using sampling quotas. Participants who completed the survey in less than one-third of the average completion time were excluded. This study followed the American Association for Public Opinion Research (AAPOR) reporting guideline for survey studies.^24^ The study protocol was approved by the institutional review board at Stanford University. This study focused on participants who reported having ever used an e-cigarette. Furthermore, we divided the sample into 2 age groups: 13 to 20 years, representing those under the legal age of e-cigarette sales (underage youth), and 21 to 24 years (young adults). Sociodemographic information on age, sex, sexual orientation, race/ethnicity, US state of residence, and self/friends adhering to stay-at-home mandates was collected (further details in eTable 1 in the Supplement).

### Measures

The survey comprised fixed-item response questions, including 39 questions asked of all participants (sociodemographic and tobacco product use). Using display logic and skip patterns depending on products ever used, participants then completed up to another 58 questions. The survey included a mixture of validated items and new items developed to capture unique participant experiences during the COVID-19 pandemic. New items were developed with input from our Youth Advisory Board (12 youth members aged 16-22 years). The survey was piloted among a small group of researchers and the Youth Advisory Board and iteratively revised.

Based on items used in previous survey studies,^11,25,26,27^ participants were asked to indicate the number of times they ever used an e-cigarette: (1) never, (2) 1 to 2 times, (3) 3 to 10 times, (4) 11 to 19 times, (5) 20 to 30 times, (6) 31 to 99 times, and (7) 100 or more times^25^ and past 30-day use of an e-cigarette, listing number of days used from 0 to 30.^11,26,27^Information on past 7-day use of e-cigarettes with the number of days used from 0 to 7^11^ was also asked separately for disposable pod-based e-cigarettes (eg, Puff Bar), pod-based e-cigarettes (eg, JUUL), or other e-cigarettes (eg, mods).

Loss of autonomy to e-cigarettes was asked through the Hooked on Nicotine Checklist (HONC), a validated scale to assess nicotine dependence,^28^ which has been used among adolescent populations^29^ and for e-cigarette products.^11^ The HONC includes 10 items that assess whether participants had tried to quit, found it difficult to quit, felt addicted, experienced cravings, and experienced withdrawal symptoms (difficulty concentrating, irritability, and nervousness). The HONC has shown agreement between test and retest when used as a diagnostic indicator (ie, selection of ≥1 symptoms) (κ = 0.74, Yules Y = 0.79, n = 74).^29^ We omitted 1 item (when you tried to stop/when you haven’t used an e-cigarette in a while, did you feel a strong need or urge to use?), resulting in 9 items for this study. Per standard HONC scoring,^28^ a response of yes to any HONC item for any e-cigarette product was coded 1, indicating nicotine dependence; all other responses were coded 0, indicating not dependent. The Cronbach α level for the 9 HONC items in our survey was 0.93.

Survey items specifically addressing changing patterns of e-cigarette use during the COVID-19 pandemic did not exist; as such, we developed these questions with input from our Youth Advisory Board and pilot testing. Participants were asked, Since the start of the COVID-19 pandemic in the US, have you changed the amount you vape? (yes/no). If participants answered yes, we asked, Which of the following best describes the change in vaping use?, with 7 response categories: (1) reduced slightly, (2) reduced the amount I vape by half, (3) quit using vapes altogether, (4) increased the amount I vape nicotine (more hours or more times in a day), (5) increased the amount I vape marijuana/THC [tetrahydrocannabinol] (more hours or more times in a day), (6) switched from vaping nicotine to other forms of nicotine, and (7) switched from vaping marijuana to other forms of marijuana. If participants checked categories indicating reduced use (reduced slightly or by half, or quit), we asked, Why did you reduce your use/switch?, with 4-response categories: (1) because I am home and parents will know, (2) because I can’t get my products, (3) because it may weaken my lungs, and (4) other, please specify. If participants checked categories indicating increased use (vaped nicotine and vaped marijuana/THC), we asked, Why did you increase your use/switch?, with 4 response categories: (1) because I am bored, (2) because I am stressed, (3) because I need a distraction, and (4) other, please specify. Those who switched use to other forms of nicotine or marijuana were asked both reasons for increasing and decreasing.

If participants reported that they switched from vaping nicotine to other forms of nicotine, we asked them to indicate which product they switched to with 5 response categories: (1) chew, (2) nicotine lozenges, (3) nicotine patches, (4) cigarette smoking, (5) nicotine gum, and (6) other, please specify. If participants indicated that they switched from vaping marijuana to other forms of marijuana, we asked them to report which product they switched to with 4 response categories: (1) edibles, (2) smoked marijuana, (3) cigarette smoking, and (4) other, please specify.

We adapted 2 questions on nicotine strength from previous surveys to provide specific response options to participants. The first of these questions was, What concentration of nicotine do you typically use in your e-cigarette? with response-categories I don’t know, 0-, 6-, 12-, 18-24-mg, and other^30^; the second question was, The nicotine content of a JUUL is 5%. Which of the following do you think best describes the amount of nicotine in a JUUL? with response categories low, medium, high, and I don’t know.^31^ Participants were then asked what nicotine strength by volume they used most often (in the context of before COVID-19 and since staying at home due to COVID-19) for disposable, pod-based, and other e-cigarettes. The question included 4 response categories: (1) less than 2.5% (<25 mg), (2) between 2.5% and 5% (approximately 25-50 mg), (3) more than 5% (>50 mg), and (4) don’t know. We then created a new variable indicating whether and how e-cigarette users changed the nicotine strength across products, coded as follows: (0) no change, (1) increase in nicotine strength, (2) decrease in nicotine strength, (3) did not know since COVID-19, (4) did not know before and knew since COVID-19, and (5) did not know before or after the COVID-19 pandemic began.

Participants were asked, How long does it usually take to finish your e-cigarette or pod?, as used in a previously published survey,^12^ both before COVID-19 and since staying at home due to COVID-19. We included 7 response categories: (1) less than a day, (2) 1 to 2 days, (3) 3 to 5 days, (4) a week (7 days), (5) 2 weeks, (6) 1 month, and (7) don’t know. We then created a new variable indicating whether and how e-cigarette users changed the time they took to finish either an e-cigarette or pod, coded as follows: (0) no change, (1) taking more time, (2) taking less time, (3) did not know since COVID-19, (4) did not know before and knew since COVID-19, and (5) did not know before or after COVID-19.

Questions on changing patterns of where youth and young adults accessed e-cigarettes during the COVID-19 pandemic did not exist; as such, we developed these questions with input from our Youth Advisory Board. Participants were asked, Since the start of the COVID-19 pandemic, what is your experience of getting vapes or vaping-related products? We provided 2 response categories: (1) it is much easier to get products and (2) it is much harder to get products. If participants found it easier to obtain the products, they were asked why it was easier with 5 response categories: (1) vape shop/dealer delivers it to my friend directly, (2) vape shop/dealer delivers it to me directly, (3) I switched to buying it online, (4) it is cheaper than before due to promotions, and (5) I use my parents’ vape. If participants responded that the products were harder to obtain, they were asked why it was harder, with 6 response categories: (1) my product is not available online, (2) I can’t go to a grocery store or gas station, (3) longer shipping times, (4) I can’t go to the vape shop, (5) it’s more expensive, and (6) restrictions on the number of products I can buy.

Participants were asked, Before COVID-19, where did you buy these product/s most often? and After stay-at-home orders due to COVID-19, if you purchased any of these product/s, where did you buy these products most often? We provided participants 10 response categories: (1) online, (2) drugstores (like Rite Aid), (3) gas station, (4) liquor store, (5) convenience store (like 7-Eleven or local mart), (6) smoke shop or vape shop, (7) medical marijuana dispensary, (8) big retail store (like Walmart), (9) supermarket (like Safeway), and (10) other, please specify. This survey question and response categories were used in a previous survey study^20^ and we adapted response categories in the National Youth Tobacco Survey question,^21^ During the past 30 days, where did you get or buy the e-cigarettes that you have used?, to provide additional categories (eg, medical marijuana dispensaries, smoke shops, and liquor stores) and description of stores to include examples (eg, Rite Aid, Walmart, and 7-11). We also asked whether age was verified at the time of purchase: When you purchased e-cigarette products, were you asked to verify your age? (yes/no/don’t know). If respondents answered yes, we asked, How was your age verified? with 5 response categories: (1) asked to upload identification (ID) as verification, (2) showed ID, (3) no age verification, (4) email login, and (5) other, please specify. Questions on age verification were developed based on studies of e-cigarette sales to underage youth, which suggested current strategies to verify age, including name, address, birth date, social security number, and other forms of ID.^32,33,34^

### Statistical Analysis

Participant characteristics were summarized for all underage youth and young adult e-cigarette users. Due to missing data in our sample, these percentages were calculated using the number of respondents as the denominator. Differences in access to e-cigarettes before and after the pandemic began were analyzed through shifts in the point-of-purchase from retail to online, online to retail, and no change among underage youth and young adults and reasons for finding it harder or easier to access e-cigarettes. Logistic regressions were conducted to assess differences in study outcomes between underage youth and young adults to provide odds ratios (ORs) and 95% CIs. In addition, univariable and multivariable logistic regressions were conducted to examine factors associated with quitting/reducing e-cigarette use since the COVID-19 pandemic began (the dominant pattern of change in youth use), including sociodemographic covariates of age, sex, identifying as lesbian, gay, bisexual, transgender, or queer, race/ethnicity, adhering to stay-at-home orders, reasons why it was harder to access products, number of times they previously used e-cigarettes, nicotine dependence, and adjusting for state-level clustering effects. Listwise deletion was used to omit cases with missing data. Additional analysis assessed whether quitting or reducing use was associated with use of specific e-cigarette device types (disposable, pod-based, and other) in the past 30 days. We used Stata, version 15.1 (StataCorp LLC) to conduct 2-tailed tests, with significance set at *P* < .05.^35^

## Results

Following the American Association for Public Opinion Research reporting guidelines for nonprobability online samples,^24^ we report that 40% of those who clicked on the survey link completed the entire survey, resulting in a 40% participation rate (ie, completion rate).^36^ We excluded from our sample 269 surveys because they did not meet Qualtrics quality checks. The final sample was 4351 participants. In this study, we focused on the 2167 participants who indicated that they had ever used an e-cigarette.

As reported in Table 1, 2167 e-cigarette users in our sample were divided into 2 age groups: younger than 21 years (underage youth, under the legal age to purchase tobacco; n = 1442) and 21 years and older (young adults; n = 725). The mean (SD) age of underage e-cigarette users was 17.51 (2.7) years and young adult e-cigarette users was 22.44 (2.9) years, with 403 of 1442 underage youth aged less than 17 years (27.9%). Our sample included 1397 females (64.5%), 723 males (33.4%), and 438 people who identified as lesbian, gay, bisexual, transgender, queer (20.2%). Participants were racially/ethnically diverse, with 319 individuals reporting as African American/Black, non-Hispanic (14.7%), 146 Asian/Pacific Islander, non-Hispanic (6.7%), 416 Hispanic, non–African American/Black (19.2%), 1102 White, non-Hispanic (50.9%), and 184 other/multiracial, non-Hispanic (8.5%). A total of 1693 of 2093 participants (80.9%) reported that they were adhering to COVID-19–related stay-at-home orders, and 1086 of 1812 (59.9%) perceived that their friends were adhering to such orders. A total of 1086 of 1992 respondents (54.5%) indicated that they used an e-cigarette in the past 30 days, 913 of 1988 individuals (46.0%) reported use within the past 7 days, 427 of 1932 respondents (22.1%) had used an e-cigarette 100 or more times, and 1331 of 2167 individuals (61.4%) were nicotine dependent.

**Table 1.** Participant Characteristics of e-Cigarette Ever-Users

Characteristic (No. of responses)	Ever-users, No. (%)		
	Total (n = 2167)	Underage (age <21 y) (n = 1442)	Young adults ( age ≥21 y) (n = 725)
Age, mean (SD), y (n = 2167)	19.17 (2.3)	17.51 (2.7)	22.44 (2.9)
Sex (n = 2166)			
Male	723 (33.4)	399 (27.7)	324 (44.7)
Female	1397 (64.5)	1006 (69.8)	391 (53.9)
Other/nonbinary	46 (2.1)	36 (2.5)	10 (1.4)
LGBTQ (n = 2166)	438 (20.2)	329 (22.8)	109 (15.0)
Race/ethnicity (n = 2167)			
African American/Black	319 (14.7)	179 (12.4)	140 (19.3)
Asian/Pacific Islander, non-Hispanic	146 (6.7)	84 (5.8)	62 (8.6)
Hispanic	416 (19.2)	262 (18.2)	154 (21.2)
White, non-Hispanic	1102 (50.9)	806 (55.9)	296 (40.8)
Other/multiracial	184 (8.5)	111 (7.7)	73 (10.1)
Past 30-d use of e-cigarettes (n = 1992)	1086 (54.5)	663 (49.6)	423 (64.5)
Past 7-d use of e-cigarettes (n = 1988)	913 (46.0)	539 (40.4)	374 (57.1)
No. of times any e-cigarette was used (n = 1932)			
1-2	472 (24.4)	331 (25.5)	141 (22.2)
3-10	432 (22.4)	284 (21.9)	148 (23.3)
11-19	210 (10.9)	124 (9.6)	86 (13.5)
20-30	208 (10.8)	132 (10.2)	76 (11.9)
31-99	183 (9.5)	117 (9.0)	66 (10.4)
≥100	427 (22.1)	309 (23.8)	118 (18.7)
Nicotine dependent (HONC score ≥1) (n = 2167)	1331 (61.4)	883 (61.2)	448 (61.8)
Adhering to stay-at-home mandate (n = 2093)	1693 (80.9)	1090 (78.9)	603 (84.6)
Friends adhering to stay-at-home mandate (n = 1812)	1086 (59.9)	633 (53.3)	453 (72.5)

Abbreviation: HONC, Hooked on Nicotine Checklist; LGBTQ, lesbian, gay, bisexual, transgender, queer.

### Change in e-Cigarette Use

Table 2 reports changes in e-cigarette use since the COVID-19 pandemic began. Among 2125 ever-e-cigarette users, 1198 (56.4%) reported that they had changed the amount they used since the start of the COVID-19 pandemic, with no significant differences between underage youth and young adults. Among those who changed their e-cigarette use, 283 of 776 underage youth (36.5%) self-reported quitting e-cigarettes since the pandemic began compared with 105 of 421 young adults (24.9%), and 239 of 776 (30.8%) of underage youth and 105 of 725 young adults (24.9%) reported reducing the amount they used e-cigarettes (slightly or by half), compared with 183 of 421 young adults (43.4%). Increased amount of nicotine in e-cigarettes during the pandemic was self-reported by 129 of 776 underage youth (16.6%) and 82 of 421 young adults (19.5%), and an increased amount of cannabis/tetrahydrocannabinol in e-cigarettes was reported by 65 of 776 underage youth (8.4%) and 29 of 421 young adults (6.9%). Further, a total of 82 e-cigarette users (6.9%) had either switched to other forms of nicotine or cannabis. Overall, 810 of 1197 (67.7%) e-cigarette users who changed their use reported quitting or reduced use of e-cigarettes (by half/slightly). Approximately 2% of both underage youth (17 [2.2%]) and young adults (10 [2.4%]) self-reported switching from e-cigarettes to other forms of nicotine, with underage youth reporting that they switched to combustible cigarettes, gum, and patches, and young adults reporting that they switched to gum and patches. Other e-cigarette users (underage youth, 43 of 776 [5.5%]; younger adults, 12 of 421 [2.8%]) said they switched from using cannabis/THC in their e-cigarettes to smoked cannabis, edible cannabis, and blunts. Table 2 also reports that 813 of 1714 participants (47.4%) reported that they did not change nicotine strength and 572 of 1840 participants (31.1%) indicated that they did not change the time used to finish their e-cigarette after the COVID-19 pandemic began. Among the 1714 participants who reported on changes, 262 individuals (15.3%) reduced nicotine strength (163 of 1167 [14.0%] underage youth and 99 of 547 [18.1%] young adult e-cigarette users) and 316 participants (17.2%) took more time to finish their e-cigarette (181 of 1247 [14.5%] underage youth and 135 of 593 [22.8%] young adults). Nicotine strength and time taken to finish different products before and after the COVID-19 pandemic began are reported in eTable 2 and eTable 3 in the Supplement.

**Table 2.** Changes in e-Cigarette Use Before and After the COVID-19 Pandemic Began^a^

Characteristic (No. of responses)	No. (%)			Unadjusted OR (95% CI)^b^
	Total sample (n = 2167)	Underage youth (age <21 y) (n = 1442)	Young adults (age ≥21 y) (n = 725)	
Participants reporting any change in the amount of e-cigarette use since COVID-19 (n = 2125)	1198 (56.4)	776 (55.1)	422 (58.9)	0.85 (0.71-1.02)
Type of change since COVID-19 (n = 1197)				
Quit using e-cigarettes	388 (32.4)	283 (36.5)	105 (24.9)	1.72 (1.32-2.25)^b^
Reduced the amount by half	232 (19.4)	143 (18.4)	89 (21.1)	0.84 (0.62-1.13)
Reduced slightly	190 (15.9)	96 (12.4)	94 (22.3)	0.49 (0.35-0.67)^b^
Increased amount of nicotine in e-cigarettes	211 (17.6)	129 (16.6)	82 (19.6)	0.82 (0.60-1.11)
Increased amount of cannabis/THC	94 (7.8)	65 (8.4)	29 (6.9)	1.23 (0.78-1.94)
Switched from e-cigarette use to other forms of nicotine	27 (2.3)	17 (2.2)	10 (2.4)	0.92 (0.41-2.02)
Combustible cigarettes	10 (37.0)	8 (47.1)	2 (20.0)	
Nicotine patches or gum	16 (59.2)	8 (47.1)	8 (80.0)	
Other	1 (3.8)	1 (5.8)	0	
Switched from using cannabis/THC in e-cigarettes to other products	55 (4.6)	43 (5.5)	12 (2.8)	1.99 (1.04-3.83)^b^
Smoked cannabis	24 (43.6)	18 (41.9)	6 (50.0)	
Edible cannabis	12 (21.8)	9 (20.9)	3 (25.1)	
Blunts	11 (20.0)	10 (23.4)	1 (8.3)	
Combustible cigarettes	4 (7.3)	3 (6.9)	1 (8.3)	
Other	4 (7.3)	3 (6.9)	1 (8.3)	
Nicotine strength before and after COVID-19 (n = 1714)				
No change	813 (47.4)	539 (46.2)	274 (50.1)	0.85 (0.69-1.04)
Reduced nicotine strength	262 (15.3)	163 (14.0)	99 (18.1)	0.73 (0.55-0.96)
Increased nicotine strength	170 (9.9)	99 (8.5)	71 (13.0)	0.62 (0.45-0.85)^b^
Did not know before or since COVID-19	300 (17.5)	233 (20.0)	67 (12.2)	1.78 (1.33-2.39)^b^
Knew nicotine strength before COVID-19 and do not know since	140 (8.2)	110 (9.3)	30 (5.5)	1.79 (1.18-2.72)^b^
Did not know nicotine strength before COVID-19 and now know	29 (1.7)	23 (2.0)	6 (1.1)	1.81 (0.73-4.47)
Time taken to finish a pod or e-cigarette before and after COVID-19 (n = 1840)				
No change	572 (31.1)	370 (29.7)	202 (34.1)	0.81 (0.66-1.01)
Taking less time to finish	348 (18.9)	230 (18.4)	118 (19.9)	0.91 (0.71-1.16)
Taking more time to finish	316 (17.2)	181 (14.5)	135 (22.8)	0.57 (0.44-0.73)^b^
Did not know before or since COVID-19	386 (20.9)	296 (23.7)	90 (15.2)	1.73 (1.34-2.25)^b^
Do not know since COVID-19	207 (11.3)	164 (13.2)	43 (7.2)	1.93 (1.36-2.75)^b^
Did not know how much was used before COVID-19 and now know	11 (0.6)	6 (0.5)	5 (0.8)	0.56 (0.17-1.87)
Reasons for decreased e-cigarette use after COVID-19 (n = 895)				
At home and parents will know	136 (15.2)	80 (13.7)	56 (17.9)	0.72 (0.50-1.05)
Cannot get products	175 (19.5)	102 (17.5)	73 (23.4)	0.69 (0.49-0.97)^b^
e-Cigarettes may weaken the lungs	224 (25.0)	132 (22.7)	92 (29.5)	0.69 (0.51-0.95)^b^
Any combination of ≥2 reasons above	287 (32.1)	213 (36.5)	74 (23.7)	1.85 (1.35-2.52)^b^
Other	73 (8.2)	56 (9.6)	17 (5.5)	1.84 (1.05-3.23)^b^
Reasons for increasing the amount of nicotine/cannabis in e-cigarettes after COVID-19 (n = 421)				
Bored	88 (20.9)	65 (24.5)	23 (14.8)	1.85 (1.09-3.13)^b^
Stressed	83 (19.7)	40 (15.0)	43 (27.8)	0.46 (0.28-0.74)^b^
Need a distraction	35 (8.3)	19 (7.1)	16 (10.4)	0.66 (0.33-1.34)
Any combination of ≥2 reasons above	201 (47.7)	131 (49.3)	70 (45.1)	1.17 (0.79-1.75)
Other	14 (3.4)	11 (4.1)	3 (1.9)	2.18 (0.60-7.95)

Abbreviations: COVID-19, coronavirus disease 2019; OR, odds ratio; THC, tetrahydrocannabinol.

^a^ Owing to missing data in our sample, percentages were calculated using the number of responses as the denominator. Therefore, the denominators using the numbers of participants in the underage (<21 years) and young adult (≥21 years) cohorts for each characteristic are as follows: participants reporting any change in the amount of e-cigarette use since the COVID-19 pandemic began (1409 underage vs 716 young adults), type of change since the pandemic began (776 underage vs 421 young adults), nicotine strength since before and after the pandemic began (1167 underage vs 547 young adults), time taken to finish a pod or e-cigarette since before and after the pandemic began (1247 underage vs 593 young adult), reasons for decreased use after the pandemic began (583 underage vs 312 young adults), and reasons for increased use after the pandemic began (266 underage vs 155 young adults).

^b^ Statistically significant at *P* < .05.

### Reasons for Changing e-Cigarette Use

Table 2 reports reasons why 895 e-cigarette users reduced their e-cigarette use since the start of the COVID-19 pandemic, including e-cigarettes may weaken their lungs (224 [25.0%]), their parents would find out that they were using e-cigarettes (136 [15.2%]), that they could not get e-cigarettes (175 [19.5%]), citing a combination of 2 or more of these reasons (287 [32.1%]), and other reasons (73 [8.2%]).Similarly, among 421 e-cigarette users citing reasons for increased use 201 e-cigarette users (47.7%) attributed their increased amount of nicotine and/or cannabis in e-cigarettes since the COVID-19 pandemic began to a combination of reasons, including boredom, stress, and need for distraction; the remaining participants cited individual reasons for increased use: boredom (88 [20.9%]), stress (83 [19.7%]), the need for a distraction (35 [8.3%], and other reasons (14 [3.4]).

### Ease or Difficulty in Accessing Products

Table 3 describes reasons why participants found it harder to access e-cigarettes since the COVID-19 pandemic began, including not being able to go to a grocery store or gas station (557 [28.7%]) or vape shop (377 [19.4%]) and longer shipping times (412 [21.2%]). More young adults than underage youth were affected by longer shipping times and products not being available online. The top reasons for finding it easy to access e-cigarettes among 261 e-cigarette users included vape shop/dealer delivers to self and friends (143 [54.8%]) and switching to online purchasing (51 [19.5%]). More than 50% of 115 underage youth who found it easier to access products were getting direct deliveries from vape shops/dealers (38 [33.0%]) or from friends who got vape shop deliveries (24 [20.9%]). Among 229 underage e-cigarette users who answered the question on whether their age was verified during recent e-cigarette purchases, 63 individuals (27.5%) reported buying e-cigarettes without any verification and 12 (5.2%) did not know if their age was verified. Among 154 of underage youth (67.3%) whose age was verified, 112 (72.7%) physically showed ID, 11 (7.1%) gave an email login, 28 (18.2%) uploaded ID information online, and 3 (1.9%) reported other. Compared with young adults, underage youth were more likely to find it difficult to get e-cigarettes because of restrictions on the number of products they could buy (unadjusted odds ratio [OR], 1.98; 95% CI, 1.35-2.90) and because they could not go to a vape shop (unadjusted OR, 1.49; 95% CI, 1.16-1.91.)

**Table 3.** Reasons Why It Is Easier or Harder to Access Products Since the COVID-19 Pandemic Began^a^

Characteristic (No. of responses)	No. (%)			Unadjusted OR (95% CI)
	Total sample (n = 2167)	Underage youth [<21] (n = 1442)	Young adults [≥21] (n = 725)	
Reasons why it is hard to get e-cigarettes (n = 1939)				
I cannot go to a grocery store or gas station	557 (28.7)	371 (29.1)	186 (28.0)	1.05 (0.85-1.29)
Longer shipping times	412 (21.2)	236 (18.5)	176 (26.5)	0.62 (0.50-0.78)^b^
I cannot go to the vape shop	377 (19.4)	274 (21.5)	103 (15.5)	1.49 (1.16-1.91)^b^
My product is not available online	250 (12.9)	151 (11.8)	99 (14.9)	0.76 (0.58-1.01)^b^
It is more expensive	177 (9.1)	113 (8.9)	64 (9.6)	0.91 (0.66-1.25)
Restrictions on number of products I can buy	166 (8.7)	130 (10.2)	36 (5.5)	1.98 (1.35-2.90)^b^
Reasons why it is easy to get e-cigarettes (n = 261)				
Vape shop/dealer delivers it to me directly	90 (34.5)	38 (33.0)	52 (35.6)	0.89 (0.53-1.49)
Vape shop/dealer delivers it to my friend directly	53 (20.3)	24 (20.9)	29 (19.9)	1.06 (0.58-1.95)
I switched to buying it online	51 (19.5)	21 (18.3)	30 (20.5)	0.86 (0.46-1.60)
It is cheaper than before due to promotions	51 (19.5)	27 (23.5)	24 (16.4)	1.56 (0.84-2.88)
I use my parents’ vape	16 (6.2)	5 (4.3)	11 (7.6)	0.55 (0.18-1.65)

Abbreviations: COVID-19, coronavirus disease 2019; OR, odds ratio.

^a^ Owing to missing data in our sample, percentages were calculated using the number of responses as the denominator. Therefore, the denominators using the numbers of participants in the underage (<21 years) and young adult (≥21 years) cohorts for each characteristic are as follows: reasons why it is hard to get e-cigarettes (1275 underage vs 664 young adults) and reasons why is it easy to get e-cigarettes (115 underage vs 146 young adults).

^b^ Statistically significant at *P* < .05.

### Change in Point-of-Purchase

Before the COVID-19 pandemic began, most participants predominantly self-reported purchasing their e-cigarettes from smoke shops, vape shops, and gas stations, followed by online and convenience stores; after the pandemic began, online purchasing was the predominant source (eTable 4 in the Supplement). As reported in Table 4, since the COVID-19 pandemic began, e-cigarette users reported shifting their point-of-purchase from one type of retail store to another (disposables, 150 of 632 [23.7%]; pod-based, 144 of 797 [18.1%]; and other e-cigarette, 125 of 560 [22.3%], ie, between 18.1% and 23.7%), shifting their point-of-purchase from retail to online (disposables, 115 of 632 [18.2%]; pod-based, 156 of 797 [19.6%]; and other e-cigarette, 111 of 560 [19.8%], ie, between 18.2% and 19.8%), shifting from online to retail (disposables, 11 of 632 [1.7%]; pod-based, 17 of 797 [2.0%]; and other e-cigarette 13 of 560 [2.3%], ie, between 1.7%-2.3%), continuing with the same retail location (disposables, 262 of 632 [41.5%]; pod-based, 344 of 797 [43.2%]; and other e-cigarette, 223 of 560 [39.8%], ie, between 39.8% and 43.2%), and continuing with the same online location (disposables, 94 of 632 [14.9%]; pod-based, 136 of 797 [17.1%]; and other e-cigarette, 88 of 560 [15.8%], ie, between 14.9% and 17.1%). Overall, 39.8% to 44.5% of e-cigarette users across different device types reported changing their point-of-purchase since the pandemic began (276 of 632 disposable e-cigarette users [43.7%]; 317 of 797 pod-based e-cigarette users [39.8%]; and 249 of 560 other e-cigarette users [44.5%]). As reported in Table 4, underage youth were 1.6 to 1.9 times (unadjusted ORs) more likely to continue with their retail location of purchasing e-cigarettes compared with young adults across disposable, pod-based, and other e-cigarettes. For pod-based e-cigarettes, underage youth were 36% less likely to change to an alternative retail location (unadjusted OR, 0.64; 95% CI, 0.45-0.92) and 77% less likely to change from online to retail sources (unadjusted OR, 0.23; 95% CI, 0.07-0.72) compared with young adults. For other e-cigarettes, underage youth were 42% less likely to change their point of purchase to an alternative retail location (unadjusted OR, 0.58; 95% CI, 0.39-0.87).

**Table 4.** Change in the Point of Purchasing e-Cigarettes Before and After COVID-19 by Product Type

Characteristic	No. (%)			Unadjusted OR (95% CI)
	Total sample	Underage youth (<21)	Young adults (≥21)	
**Disposables**				
No.	632	350	282	
No change in retail location	262 (41.5)	165 (47.2)	97 (34.5)	1.70 (1.23-2.34)^a^
No change in online location	94 (14.9)	45 (12.8)	49 (17.4)	0.70 (0.45-1.08)
Changed from retail to online	115 (18.2)	61 (17.4)	54 (19.1)	0.89 (0.59-1.33)
Changed from retail to other type of retail	150 (23.7)	74 (21.2)	76 (26.9)	0.72 (0.50-1.05)
Changed from online to retail	11 (1.7)	5 (1.4)	6 (2.1)	0.66 (0.20-2.21)
**Pod-based e-cigarettes**				
No.	797	447	350	
No change in retail location	344 (43.2)	216 (48.3)	128 (36.6)	1.62 (1.21-2.15)^a^
No change in online location	136 (17.1)	76 (17.0)	60 (17.1)	0.99 (0.68-1.43)
Changed from retail to online	156 (19.6)	83 (18.6)	73 (20.9)	0.86 (0.61-1.22)
Changed from retail to other type of retail	144 (18.1)	68 (15.2)	76 (21.7)	0.64 (0.45-0.92)^a^
Changed from online to retail	17 (2.0)	4 (0.8)	13 (3.7)	0.23 (0.07-0.72)^a^
**Other e-cigarettes**				
No.	560	308	252	
No change in retail location	223 (39.8)	144 (46.7)	79 (31.3)	1.92 (1.35-2.72)^a^
No change in online location	88 (15.8)	44 (14.4)	44 (17.5)	0.78 (0.49-1.24)
Changed from retail to online	111 (19.8)	58 (18.8)	53 (21.0)	0.87 (0.57-1.32)
Changed from retail to other type of retail	125 (22.3)	56 (18.2)	69 (27.4)	0.58 (0.39-0.87)^a^
Changed from online to retail	13 (2.3)	6 (1.9)	7 (2.8)	0.69 (0.23-2.09)

Abbreviations: COVID-19, coronavirus disease 2019; OR, odds ratio.

^a^ Statistically significant at *P* < .05.

### Factors Associated With Quitting or Reducing e-Cigarette Use 

As reported in Table 5, quitting or reducing e-cigarette use since the COVID-19 pandemic began was 1.9 times more likely among e-cigarette users who were Asian/Pacific Islander (adjusted OR [aOR], 1.88; 95% CI, 1.10-3.22), 1.6 times more likely among other/multiracial, non-Hispanic individuals (aOR, 1.60; 95% CI, 1.04-2.47), and 1.5 times more likely among those who were adhering to stay-at-home mandates (aOR, 1.49; 95% CI, 1.06-2.08). e-Cigarette users were 31% less likely to quit if they experienced longer shipping times (aOR, 0.69; 95% CI, 0.49-0.95), 52% less likely to quit if they used an e-cigarette 11 to 99 times (aOR, 0.48; 95% CI, 0.30-0.78), 68% less likely if they used an e-cigarette 100 or more times (aOR, 0.32; 95% CI, 0.20-0.51), and 51% less likely to quit if they were nicotine dependent (aOR, 0.49; 95% CI, 0.35-0.70). Underage youth were not more likely to quit or reduce e-cigarette use compared with young adults, despite finding it harder to access their products. We included device types used in the past 30 days to this model in an additional analysis and observed that past 30-day pod-based e-cigarette-users were 47% less likely to quit or reduce their use (aOR, 5.3; 95% CI, 0.31-0.88), and Asian/Pacific Islander, non-Hispanic (aOR, 2.11; 95% CI, 1.15-3.85), other/multiracial, non-Hispanic (aOR, 2.77; 95% CI, 1.21-6.34) and Hispanic, non–African American/Black (aOR, 2.48; 95% CI, 1.41-4.34) participants were more than 2 times more likely to quit or reduce their use; and participants who had previously used e-cigarettes more than 100 times were 59% less likely to quit/reduce use (aOR, 0.41; 95% CI, 0.20-0.83).

**Table 5.** Association Between Reduced e-Cigarette Use During COVID-19 and Other Factors

Characteristic	Quitting or reduced e-cigarette use [n = 991], OR (95% CI)	
	Unadjusted	Adjusted
Legal age of purchasing tobacco, including e-cigarettes, y		
13-20 (underage youth)	0.94 (0.73-1.22)	1.07 (0.77-1.49)
21-24 (young adult)	1 [Reference]	1 [Reference]
Sex		
Male	1.06 (0.82-1.37)	0.98 (0.77-1.25)
Other	1.04 (0.46-2.35)	1.10 (0.47-2.57)
Female	1 [Reference]	1 [Reference]
LGBTQ		
Yes	0.76 (0.57-2.54)	0.80 (0.57-1.11)
No	1 [Reference]	1 [Reference]
Race/ethnicity		
African American/Black, non-Hispanic	1.56 (1.10-2.21)^a^	1.20 (.76-1.88)
Asian/ Pacific Islander	2.35 (1.39-3.96)^a^	1.88 (1.10-3.22)^a^
Other/multiracial, non-Hispanic	1.62 (1.17-2.25)^a^	1.60 (1.04-2.47)^a^
Hispanic, non-African American/Black	1.13 (.74-1.71)	1.40 (0.85-2.32)
White, non-Hispanic	1 [Reference]	1 [Reference]
Adhering to stay-at-home mandate		
Yes	1.55 (1.12-2.15)^a^	1.49 (1.06-2.08)^a^
No	1 [Reference]	1 [Reference]
Harder to get products		
Longer shipping times	0.84 (0.59-1.20)	0.69 (0.49-0.95)^a^
I can’t go to the vape shop	0.89 (0.62-1.28)	0.84 (0.63-1.14)
My product is not available online	1.51 (0.97-2.34)	1.34 (0.78-2.31)
It’s more expensive	0.77 (0.48-1.22)	0.71 (0.44-1.16)
Restrictions on number of products I can buy	1.15 (0.69-2.60)	1.05 (0.68-1.64)
I can’t go to a grocery store or gas station	1 [Reference]	1 [Reference]
No. of times an e-cigarette was used		
11-99	0.44 (0.32-0.61)^a^	0.48 (0.30-0.78)^a^
≥100	0.24 (0.17-0.34)^a^	0.32 (0.20-0.51)^a^
1-10	1 [Reference]	1 [Reference]
Nicotine dependence		
Dependent (any answer as yes to HONC)	0.45 (0.33-0.61)^a^	0.49 (0.35-0.70)^a^
Not dependent (all other answers)	1 [Reference]	1 [Reference]

Abbreviations: COVID-19, coronavirus disease 2019; HONC, Hooked on Nicotine Checklist; LGBTQ, lesbian, gay, bisexual, transgender, queer; OR, odds ratio.

^a^ Statistically significant at *P* > .05.

## Discussion

Our study from a national convenience sample of underage youth and young adult ever e-cigarette users is, to our knowledge, among the first to describe changes in e-cigarette use, access to e-cigarettes, and reasons for these changes since the COVID-19 pandemic began. Findings show that 67.7% of all e-cigarette users who changed their use reported quitting or reduced use of e-cigarettes, 17.6% increased the amount of nicotine used in e-cigarettes, 7.8% increased the amount of cannabis used in e-cigarettes, and 6.9% switched to other nicotine or cannabis products. Self-reported access to e-cigarettes changed during the COVID-19 pandemic for 39.8%-44.5% of e-cigarette users, with 18.1% to 23.7% switching to alternative retail locations, 18.2% to 19.8% of e-cigarette users switching their point-of-purchase from retail to online stores, and approximately 2% switching from online to retail stores, depending on the product. Understanding patterns of e-cigarette use during the COVID-19 pandemic has the potential to inform prevention efforts to reduce e-cigarette use and associated lung and respiratory illnesses that may be associated with an increased risk of COVID-19 among underage youth and young adults.^17,18,19^

Our findings suggest that vape shops and online platforms are routinely selling to underage youth during this pandemic. Underage youth were likely to face access-related issues owing to restrictions associated with the COVID-19 pandemic because their parents were likely to be home and they could not easily go to stores, in addition to the fact that they were under the legal age of sale for tobacco. Nevertheless, there was no significant difference in quitting or reduced use between underage youth and young adults. We found that nicotine dependence and having used e-cigarettes a large number of times (>10) were associated factors that made it less likely for underage youth to quit or reduce their use of e-cigarettes. Longer shipping times made it less likely for participants to report quitting or reduced use, potentially because they anticipated future deliveries. We also found that race/ethnicity and adhering to stay-at-home mandates were significant factors associated with quitting or reduced use. Additional analysis showed that e-cigarette users who reported past 30-day use of pod-based e-cigarettes were 47% less likely to reduce e-cigarette use or quit. Underage youth who found it easier (not harder) to access e-cigarettes during the pandemic reported getting them online and from vape shops/dealers that delivered directly to them or to their friends. These findings underscore the need to effectively verify age online and in person.

Findings in this study necessitate further examination for several reasons. First, despite some e-cigarette companies indicating that they have enhanced their online age-verification methods to prevent youth access, our findings show that still over a quarter of youth are not being asked to verify their age (from among those who answered the question). Underage youth who turn to online purchases of e-cigarettes during the pandemic in lieu of their previous sources may continue to use these easy-access means, making online prohibitions or age verification even more essential. The US Food and Drug Administration should use its authority to prevent online sales of e-cigarettes to underage youth^37^ and deny marketing authorization for e-cigarette companies whose premarket tobacco product applications^38^ do not prove that their online age-verification tools work to keep underage youth from purchasing e-cigarettes. Regardless of federal action, states and localities can also require effective age verification for online e-cigarette sales and rescind licenses and/or assess penalties for companies that allow underage online purchases. In addition, states and localities can enact and enforce laws, such as in California, that prohibit sales and delivery to persons under age 21 years by “public or private postal services,” and require age verification and signatures.^39^

Second, since participants who were less likely to report quitting or reducing e-cigarette use had previously used a large number of times and are nicotine dependent, cessation resources, including nicotine step-down approaches, are needed. In this study, sheltering-in-place policies that may have limited access to retail store purchases may have helped facilitate quitting or reduced use among both underage youth and young adults. Self-reported reducing and quitting behaviors among some e-cigarette users during the pandemic are positive, especially given emerging evidence that COVID-19 may be associated with inhaled tobacco use, potentially due to a weakened respiratory system and compromised immune system.^17,18,19^ However, we do not know about quitting-related nicotine withdrawal symptoms, physical and mental health challenges, or whether switching to other products will gradually reduce nicotine use or help cope with nicotine withdrawal. Furthermore, we do not know if quit attempts will be sustained after the COVID-19 pandemic. Additional research is needed to guide e-cigarette users who are quitting or reducing their use and to inform cessation interventions. These findings may further support US Food and Drug Administration regulations requiring standardized nicotine delivery in tobacco products so that e-cigarettes are less addictive and easier for underage youth to quit.^40^

In addition, it is notable that the least-cited reason for decreased e-cigarette use after the COVID-19 pandemic began was that “parents will know,” which suggests that the small and concealable design of e-cigarettes (often replicating flash drives or highlighters) allow underage youth to continue to hide and use e-cigarettes at home, even with family members nearby. The US Food and Drug Administration has the authority to regulate marketing that targets youth^37^ and to adopt tobacco product standards “for the protection of the public health,” including “provisions respecting the construction, components…and properties of the tobacco product”^40^ relating to the overall design and concealability of e-cigarettes. In July 2020, the US Food and Drug Administration issued warning letters to companies whose electronic nicotine delivery systems products resembling watches were “designed to conceal a tobacco product from parents, teachers, or other adults.”^41,42,43^ Furthermore, because evidence shows that secondhand e-cigarette aerosol contains toxic compounds^44,45,46^ and it is unknown whether underage youth are using these products around parents and family members at home, prevention education may help parents and others to recognize devices and reduce involuntary exposure to aerosol.

### Limitations

The study has limitations. Findings from this national convenience sample are not generalizable; however, because we have a well-balanced sample on sex and race/ethnicity, our findings are suggestive of patterns among the wider US population of e-cigarette users aged 13 to 24 years. Our entire analysis focused on patterns of use and access among e-cigarette users and may not be comparable with studies on populations that include nonusers. The mean age of underage youth (17 years) in this study is closer to the age of a young adult population, possibly implying that their relationship with parents at home, access to credit cards, and independence in decision-making related to staying at home may have been more similar to the young adult vs children aged 13 years in our study. In addition, in this study, we did not account for state- and county-level regulation and enforcement of tobacco policies as other potential confounders. Self-reported data may have also been affected by recall bias, as several questions related to the time period before and since the COVID-19 pandemic began. Fewer participants answered the question on reasons why they found it easier to access e-cigarettes since the pandemic began. We did not assess whether e-cigarette users were using the products with friends in a social setting, thereby making it difficult to assess whether they were unable to continue using during the COVID-19 pandemic for these reasons as they were accustomed to using with others. Since completion of this study, some stay-at-home orders have been lifted and some schools and businesses have reopened across many US locations. Further research is needed to assess the pattern of e-cigarette use and access in subsequent stages of the COVID-19 pandemic.

## Conclusions

In this survey study of US adolescents and young adults during the COVID-19 pandemic, more e-cigarette users self-reported quitting or reducing e-cigarette use than those increasing e-cigarette use or switching to other nicotine and cannabis products. e-Cigarette users self-reported that during the COVID-19 pandemic they changed their point-of-purchase from retail stores, such as vape shops and gas stations, to purchasing from alternative retail locations and online. We found that in-store and online unregulated sales to underage youth continued through this stage of the pandemic. Despite the fact that underage youth likely experienced greater restrictions on their movement and use of e-cigarettes in their home, we observed no significant difference in their quitting behavior compared with young adults.

## Notes

Supplement. **eTable 1.** Measures on Sociodemographic Information **eTable 2.** Nicotine Strength Used Before and After COVID-19 **eTable 3.** Time Taken to Finish an e-Cigarette or Pod Before and After COVID-19 **eTable 4.** Percentage of Participants Above and Below the Legal Age Purchasing e-Cigarettes From Locations Before and After COVID-19

## References

[1] Further Consolidated Appropriations Act, 2020. In: Sec. 603, Minimum age of sale of tobacco products (December 16, 2019):1492-1503.

[2] Centers for Disease Control and Prevention Quick facts on the risks of e-cigarettes for kids, teens, and young adults. Updated February 3, 2020. Accessed July 1, 2020. https://www.cdc.gov/tobacco/basic_information/e-cigarettes/Quick-Facts-on-the-Risks-of-E-cigarettes-for-Kids-Teens-and-Young-Adults.html

[3] Cullen KA, Gentzke AS, Sawdey MD, e-Cigarette use among youth in the United States, 2019. JAMA. 2019;322(21):2095-2103. doi:10.1001/jama.2019.18387 31688912 PMC6865299

[4] Villarroel MA, Cha AE, Vahratian A Electronic cigarette use among US adults, 2018: NCHS data brief No.365. National Center for Health Statistics, Centers for Disease Control and Prevention, US Department of Health and Human Services. Published 2020. Accessed May 24, 2020. https://stacks.cdc.gov/view/cdc/87918

[5] Kim M, Popova L, Halpern-Felsher B, Ling PM Effects of e-cigarette advertisements on adolescents’ perceptions of cigarettes. Health Commun. 2019;34(3):290-297. doi:10.1080/10410236.2017.1407230 29236550 PMC5999542

[6] Kim M, Ling PM, Ramamurthi D, Halpern-Felsher B Youth’s perceptions of e-cigarette advertisements with cessation claims. Tob Regul Sci. 2019;5(2):94-104. doi:10.18001/TRS.5.2.1 31840040 PMC6910223

[7] McKelvey K, Halpern-Felsher B Youth say flavored e-cigarette ads are for them. J Adolesc Health. 2018;62(2):S136-S137. doi:10.1016/j.jadohealth.2017.11.278 30314868

[8] Jackler RK, Ramamurthi D Unicorns cartoons: marketing sweet and creamy e-juice to youth. Tob Control. 2017;26(4):471-475. doi:10.1136/tobaccocontrol-2016-053206 27543562

[9] Cullen KA, Liu ST, Bernat JK, Flavored tobacco product use among middle and high school students—United States, 2014-2018. MMWR Morb Mortal Wkly Rep. 2019;68(39):839-844. doi:10.15585/mmwr.mm6839a2 31581163 PMC6776376

[10] Kong G, Morean ME, Cavallo DA, Camenga DR, Krishnan-Sarin S Reasons for electronic cigarette experimentation and discontinuation among adolescents and young adults. Nicotine Tob Res. 2015;17(7):847-854. doi:10.1093/ntr/ntu257 25481917 PMC4674436

[11] McKelvey K, Baiocchi M, Halpern-Felsher B Adolescents’ and young adults’ use and perceptions of pod-based electronic cigarettes. JAMA Netw Open. 2018;1(6):e183535-e183535. doi:10.1001/jamanetworkopen.2018.3535 30646249 PMC6324423

[12] McKelvey K, Halpern-Felsher B How and why California young adults are using different brands of pod-type electronic cigarettes in 2019: implications for researchers and regulators. J Adolesc Health. 2020;67(1):46-52. doi:10.1016/j.jadohealth.2020.01.017 32192827 PMC7311231

[13] Ramamurthi D, Chau C, Jackler RK JUUL and other stealth vaporisers: hiding the habit from parents and teachers. Tob Control. 2018;28(6):610-616. doi:10.1136/tobaccocontrol-2018-054455 30219794

[14] Ghosh A, Coakley RD, Ghio AJ, Chronic e-cigarette use increases neutrophil elastase and matrix metalloprotease levels in the lung. Am J Respir Crit Care Med. 2019;200(11):1392-1401. doi:10.1164/rccm.201903-0615OC 31390877 PMC6884043

[15] McConnell R, Barrington-Trimis JL, Wang K, Electronic cigarette use and respiratory symptoms in adolescents. Am J Respir Crit Care Med. 2017;195(8):1043-1049. doi:10.1164/rccm.201604-0804OC 27806211 PMC5422647

[16] Wills TA, Pagano I, Williams RJ, Tam EK e-Cigarette use and respiratory disorder in an adult sample. Drug Alcohol Depend. 2019;194:363-370. doi:10.1016/j.drugalcdep.2018.10.004 30472577 PMC6312492

[17] National Institute of Drug Abuse COVID-19: potential implications for individuals with substance use disorders. Published April 6, 2020. Accessed May 20, 2020. https://www.drugabuse.gov/about-nida/noras-blog/2020/04/covid-19-potential-implications-individuals-substance-use-disorders

[18] Javelle E Electronic cigarette and vaping should be discouraged during the new coronavirus SARS-CoV-2 pandemic. Arch Toxicol. 2020;94(6):2261-2262. doi:10.1007/s00204-020-02744-z 32303807 PMC7165073

[19] Gaiha SM, Cheng J, Halpern-Felsher B Association between youth smoking, electronic cigarette use, and COVID-19. J Adolesc Health. 2020;67(4):519-523. doi:10.1016/j.jadohealth.2020.07.002 32798097 PMC7417895

[20] Meyers MJ, Delucchi K, Halpern-Felsher B Access to tobacco among California high school students: the role of family members, peers, and retail venues. J Adolesc Health. 2017;61(3):385-388. doi:10.1016/j.jadohealth.2017.04.012 28712593 PMC5610576

[21] Centers for Disease Control and Prevention Historical NYTS data and documentation. Office on Smoking and Health, National Center for Chronic Disease Prevention and Health Promotion. Updated December 23, 2019. Accessed September 2, 2020. https://www.cdc.gov/tobacco/data_statistics/surveys/nyts/data/index.html

[22] Truth Initiative Where are kids getting JUUL? Published May 29, 2018. Accessed June 12, 2020. https://truthinitiative.org/news/where-are-kids-getting-juul

[23] Qualtrics. ESOMAR 28: 28 Questions to help research buyers of online samples. Updated June 20, 2014. Accessed September 24, 2020. https://success.qualtrics.com/rs/qualtrics/images/ESOMAR%2028%202014.pdf

[24] American Association for Public Opinion Research Standard Definitions: Final Dispositions of Case Codes and Outcome Rates for Surveys. 9th ed American Association for Public Opinion Research; 2016.

[25] Gorukanti A, Delucchi K, Ling P, Fisher-Travis R, Halpern-Felsher B Adolescents’ attitudes towards e-cigarette ingredients, safety, addictive properties, social norms, and regulation. Prev Med. 2017;94:65-71. doi:10.1016/j.ypmed.2016.10.019 27773711 PMC5373091

[26] Krishnan-Sarin S, Jackson A, Morean M, E-cigarette devices used by high-school youth. Drug Alcohol Depend. 2019;194:395-400. doi:10.1016/j.drugalcdep.2018.10.022 30497057 PMC6312472

[27] Cullen KA, Ambrose BK, Gentzke AS, Apelberg BJ, Jamal A, King BA Notes from the field: use of electronic cigarettes and any tobacco product among middle and high school students—United States, 2011-2018. MMWR Morb Mortal Wkly Rep. 2018;67(45):1276-1277. doi:10.15585/mmwr.mm6745a5 30439875 PMC6290807

[28] DiFranza JR, Savageau JA, Fletcher K, Measuring the loss of autonomy over nicotine use in adolescents: the DANDY (Development and Assessment of Nicotine Dependence in Youths) study. Arch Pediatr Adolesc Med. 2002;156(4):397-403. doi:10.1001/archpedi.156.4.397 11929376

[29] Wheeler KC, Fletcher KE, Wellman RJ, Difranza JR Screening adolescents for nicotine dependence: the Hooked on Nicotine Checklist. J Adolesc Health. 2004;35(3):225-230. doi:10.1016/S1054-139X(03)00531-7 15313504

[30] Morean ME, Kong G, Cavallo DA, Camenga DR, Krishnan-Sarin S Nicotine concentration of e-cigarettes used by adolescents. Drug Alcohol Depend. 2016;167(167):224-227. doi:10.1016/j.drugalcdep.2016.06.031 27592270 PMC5158305

[31] Morean ME, Bold KW, Kong G, Adolescents’ awareness of the nicotine strength and e-cigarette status of JUUL e-cigarettes. Drug Alcohol Depend. 2019;204:107512. doi:10.1016/j.drugalcdep.2019.05.032 31487572 PMC6878179

[32] Williams RS, Derrick J, Ribisl KM Electronic cigarette sales to minors via the internet. JAMA Pediatr. 2015;169(3):e1563-e1563. doi:10.1001/jamapediatrics.2015.63 25730697 PMC4408777

[33] Williams RS, Derrick J, Liebman AK, LaFleur K, Ribisl KM Content analysis of age verification, purchase and delivery methods of internet e-cigarette vendors, 2013 and 2014. Tob Control. 2018;27(3):287-293. doi:10.1136/tobaccocontrol-2016-053616 28484040 PMC5677573

[34] Unger JB, Bartsch L Exposure to tobacco websites: associations with cigarette and e-cigarette use and susceptibility among adolescents. Addict Behav. 2018;78:120-123. doi:10.1016/j.addbeh.2017.11.012 29154150 PMC5783751

[35] Stata Statistical Software: Release 15. College Station, TX: StataCorp LLC; 2017.

[36] Callegaro M, DiSogra C. Computing response metrics for online panels. Public Opinion Q. 2008;72(5):1008-1032. doi:10.1093/poq/nfn065

[37] Family Smoking Prevention and Tobacco Control Act, 906(d), Pub. L. 111-31, 21 USC 387f. Published June 22, 2009. Accessed July 1, 2020. https://www.govinfo.gov/content/pkg/PLAW-111publ31/pdf/PLAW-111publ31.pdf

[38] Food and Drug Administration Enforcement priorities for electronic nicotine delivery systems (ENDS) and other deemed products on the market without premarket authorization (revised): guidance for industry. Updated April 2020. Accessed July 1, 2020. https://www.fda.gov/media/133880/download

[39] State of California Business & Professions Code section 22963. In:2020.

[40] Family Smoking Prevention and Tobacco Control Act, Section 907(a), Pub. L. 111-31, 21 USC 387g, (2009).

[41] Food and Drug Administration Warning letter: Provape Enterprise, Inc: MARCS-CMS 608866. Published July 31, 2020. Accessed September 24, 2020. https://www.fda.gov/inspections-compliance-enforcement-and-criminal-investigations/warning-letters/provape-enterprise-inc-608866-07312020

[42] Food and Drug Administration Warning letter: Vapor Plus OK: MARCS-CMS 608888. Published July 31, 2020. Accessed September 24, 2020. https://www.fda.gov/inspections-compliance-enforcement-and-criminal-investigations/warning-letters/vapor-plus-ok-608888-07312020

[43] Food and Drug Administration Warning letter: Ejuicesteals.com: MARCS-CMS 608397. Published July 31, 2020. Accessed September 24, 2020. https://www.fda.gov/inspections-compliance-enforcement-and-criminal-investigations/warning-letters/ejuicestealscom-608397-07312020

[44] Czogala J, Goniewicz ML, Fidelus B, Zielinska-Danch W, Travers MJ, Sobczak A Secondhand exposure to vapors from electronic cigarettes. Nicotine Tob Res. 2014;16(6):655-662. doi:10.1093/ntr/ntt203 24336346 PMC4565991

[45] Drummond MB, Upson D Electronic cigarettes: potential harms and benefits. Ann Am Thorac Soc. 2014;11(2):236-242. doi:10.1513/AnnalsATS.201311-391FR 24575993 PMC5469426

[46] Goniewicz ML, Knysak J, Gawron M, Levels of selected carcinogens and toxicants in vapour from electronic cigarettes. Tob Control. 2014;23(2):133-139. doi:10.1136/tobaccocontrol-2012-050859 23467656 PMC4154473

