# Supplementary material for: Underage Youth and Young Adult e-Cigarette Use and Access Before and During the Coronavirus Disease 2019 Pandemic
Source: JAMA Netw Open. 2020 Dec 3;3(12):e2027572. doi: 10.1001/jamanetworkopen.2020.27572 (PMC7716191; doi:10.1001/jamanetworkopen.2020.27572)
Supplement: Supplement. — eTable 1. Measures on Sociodemographic Information eTable 2. Nicotine Strength Used Before and After COVID-19 eTable 3. Time Taken to Finish an e-Cigarette or Pod Before and After COVID-19 eTable 4. Percentage of Participants Above and Below the Legal Age Purchasing e-Cigarettes From Locations Before and After COVID-19 [file jamanetwopen-e2027572-s001.pdf]

Supplementary Online Content

Gaiha SM, Lempert LK, Halpern-Felsher B. Underage youth and young adult e-cigarette use and access before and during the coronavirus disease 2019 pandemic. *JAMA Netw Open*. 2020;3(12):e2027572. doi:10.1001/jamanetworkopen.2020.27572

eTable 1. Measures on Sociodemographic Information

eTable 2. Nicotine Strength Used Before and After COVID-19

eTable 3. Time Taken to Finish an e-Cigarette or Pod Before and After COVID-19

eTable 4. Percentage of Participants Above and Below the Legal Age Purchasing e-Cigarettes From Locations Before and After COVID-19

This supplementary material has been provided by the authors to give readers additional information about their work.

© 2020 Gaiha SM et al. *JAMA Network Open.*

eTable 1. Measures on sociodemographic information

Measure	Survey question	Response type and code
Socio-demographic information		
Age	How old are you today?	Numeric (age between 13-24 years). Responses 13-17 categorized as "Adolescents;" 18-21 as "Young adults" and 22-24 as "Adults."
Sex	Do you identify as… 1) Female; 2) Male, 3) Non-Binary/ Other; 4) Choose to not specify/indicate.	Categorial.<br>Females were coded "1," males as "2" and all others as "3."
LGBTQ	Do you consider yourself to be: 1) Heterosexual or straight; 2) LGBTQ+ , 3) Other, please specify_________	Categorial.<br>Response yes to 2) LGBTQ+ were coded "1" and all others as "0."
Race/ethnicity	A. What is your ethnicity?<br>1) Hispanic or Latino; 2) Not Hispanic or Latino<br>B. What is your race? Choose one or more if applicable. 1) American Indian or Alaska Native; 2) Asian; 3) Black or African American ; 4) Native Hawaiian or Other Pacific Islander; 5) White; 6) More than one race; 7) Prefer not to answer	Categorial.<br>Responses to A and B were coded as "0" for White, non-Hispanic; "1" for AA/Black, non-Hispanic; "2" for Asian/ Native Hawaiian or Pacific Islander, non-Hispanic; "3" Hispanic, non-AA/black and "4" for Other/multiracial, non-Hispanic. Prefer not to answer were included with 4, Other/ multiracial.
State and region	In what state or U.S. territory do you live?	Categorical; Responses were coded in to 5 regions based on US Census data.
Complying with Stay-at-home mandate	Are you completely complying with shelter-in-place or stay at home orders?<br>1) Yes, 2) No, 3) Don't know	Dichotomous (Yes/ No).<br>Yes was coded "1" and No was coded "0." No participants answered 3).
Friends complying with stay-at-home mandate	Are your friends completely complying with shelter-in-place or stay at home orders?<br>1) Yes, 2) No, 3) Don't know	Dichotomous (Yes/ No).<br>Yes was coded "1" and No was coded "0." No participants answered 3).

© 2020 Gaiha SM et al. *JAMA Network Open.*

eTable 2. Nicotine strength used before and after COVID-19, no. (%)

Nicotine strength before COVID-19	Nicotine strength after COVID-19				
	**Less than 2.5% (less than 25mg)**	**Between 2.5% and 5% (about 25-50 mg)**	**More than 5% (more than 50mg)**	**Don't know**	**Total**
**Disposable pod-based e-cigarettes**					
Less than 2.5% (less than 25mg)	137 (43.8)	35 (10.3)	16 (8.0)	30 (12.4)	218 (19.9)
Between 2.5% and 5% (about 25-50 mg)	127 (40.6)	266 (78.2)	58 (29.0)	42 (17.4)	493 (45.1)
More than 5% (more than 50mg)	38 (12.1)	35 (10.3)	121 (60.5)	20 (8.3)	214 (19.5)
Don't know	11 (3.5)	4 (1.2)	5 (2.5)	150 (61.9)	170 (15.5)
Total	313 (100.0)	340 (100.0)	200 (100.0)	242 (100.0)	1095 (100.0)
**Pod-based e-cigarettes**					
Less than 2.5% (less than 25mg)	96 (54.8)	15 (9.9)	5 (5.9)	29 (14.3)	145 (23.6)
Between 2.5% and 5% (about 25-50 mg)	50 (28.6)	111 (73.5)	22 (25.9)	19 (9.4)	202 (32.9)
More than 5% (more than 50mg)	22 (12.6)	23 (15.2)	55 (64.7)	12 (5.9)	112 (18.2)
Don't know	7 (4.0)	2 (1.3)	3 (3.5)	143 (70.4)	155 (25.2)
Total	175 (100.0)	151 (100.0)	85 (100.0)	203 (100.0)	614 (100.0)
**Other e-cigarettes**					
Less than 2.5% (less than 25mg)	74 (66.1)	16 (15.8)	10 (13.3)	16 (13.2)	116 (28.4)
Between 2.5% and 5% (about 25-50 mg)	23 (20.5)	71 (70.3)	19 (25.3)	14 (11.6)	127 (31.0)
More than 5% (more than 50mg)	11 (9.8)	13 (12.9)	43 (57.3)	9 (7.4)	76 (18.6)
Don't know	4 (3.6)	1 (1.0)	3 (4.0)	82 (67.8)	90 (22.9)
Total	112 (100.0)	101 (100.0)	75 (100.0)	121 (100.0)	409 (100.0)

© 2020 Gaiha SM et al. *JAMA Network Open.*

eTable 3. Time taken to finish an e-cigarette or pod before and after COVID-19, no. (%)

Time taken to finish before COVID-19	Time taken to finish after COVID-19							
	<1 day	1-2 days	3-5 days	1 week	2 weeks	1 month	Don’t know	Total
**Disposable pod-based e-cigarettes**								
**<1 day**	46 (42.6)	16 (8.2)	7 (3.4)	2 (1.3)	4 (4.1)	5 (4.8)	9 (2.8)	89 (7.5)
**1-2 days**	39 (36.1)	76 (39.2)	33 (16.0)	15 (10.1)	6 (6.1)	3 (2.8)	17 (5.2)	189 (15.9)
**3-5 days**	14 (12.9)	83 (42.8)	107 (51.9)	37 (24.8)	16 (16.3)	13 (12.4)	29 (8.9)	299 (25.2)
**1 week**	6 (5.6)	13 (6.7)	42 (20.4)	56 (37.6)	27 (27.5)	12 (11.4)	27 (8.3)	183 (15.4)
**2 weeks**	2 (1.8)	3 (1.5)	12 (5.8)	33 (22.1)	30 (30.6)	13 (12.4)	36 (11.0)	129 (10.9)
**1 month**	1 (0.9)	0 (0.0)	3 (1.5)	3 (2.0)	14 (14.3)	56 (53.3)	32 (9.8)	109 (9.2)
**Don’t know**	0 (0.0)	3 (1.5)	2 (0.9)	3 (2.0)	1 (1.0)	3 (2.9)	176 (53.9)	188 (15.8)
**Total**	108 (100.0)	194 (100.0)	206 (100.0)	149 (100.0)	98 (100.0)	105 (100.0)	326 (100.0)	1186 (100.0)
**Pod-based e-cigarettes**								
**<1 day**	56 (43.4)	11 (6.2)	9 (3.9)	1 (0.6)	1 (0.9)	2 (1.2)	14 (2.4)	94 (6.1)
**1-2 days**	46 (35.7)	86 (48.6)	38 (16.5)	17 (10.3)	6 (5.9)	5 (3.1)	27 (4.7)	225 (14.6)
**3-5 days**	21 (16.3)	66 (37.3)	111 (48.3)	35 (21.2)	13 (12.9)	14 (8.6)	34 (5.9)	294 (19.1)
**1 week**	2 (1.5)	9 (5.1)	51 (22.2)	77 (46.7)	19 (18.8)	16 (9.8)	41 (7.2)	215 (13.9)
**2 weeks**	1 (0.8)	4 (2.3)	15 (6.5)	31 (18.8)	51 (50.5)	31 (19.0)	32 (5.6)	165 (10.7)
**1 month**	2 (1.5)	1 (0.5)	3 (1.3)	3 (1.8)	11 (10.9)	90 (55.2)	48 (8.4)	158 (10.3)
**Don’t know**	1 (0.8)	0 (0.0)	3 (1.3)	1 (0.6)	0 (0.0)	5 (3.1)	377 (65.8)	387 (25.2)
**Total**	129 (100.0)	177 (100.0)	230 (100.0)	165 (100.0)	101 (100.0)	163 (100.0)	573 (100.0)	1538 (100.0)
**Other e-cigarettes**								
**<1 day**	42 (45.2)	9 (7.5)	6 (3.5)	6 (4.6)	0 (0.0)	2 (1.7)	3 (0.8)	68 (6.2)
**1-2 days**	31 (33.3)	56 (46.7)	33 (19.4)	12 (9.2)	3 (3.6)	4 (3.4)	7 (1.8)	146 (13.3)
**3-5 days**	13 (13.9)	38 (31.7)	80 (47.1)	20 (15.4)	7 (8.4)	4 (3.4)	22 (5.7)	184 (16.7)
**1 week**	3 (3.2)	10 (8.3)	37 (21.8)	62 (47.7)	16 (19.3)	11 (9.4)	15 (3.9)	154 (14.0)
**2 weeks**	0 (0.0)	3 (2.5)	13 (7.6)	27 (20.8)	37 (44.6)	12 (10.3)	20 (5.2)	112 (12.6)
**1 month**	1 (1.1)	3 (2.5)	0 (0.0)	2 (1.5)	18 (21.7)	80 (68.4)	35 (9.0)	139 (12.6)
**Don’t know**	3 (3.2)	1 (0.8)	1 (0.6)	1 (0.8)	2 (2.4)	4 93.4)	285 (73.6)	297 (27.0)
**Total**	93 (100.0)	120 (100.0)	170 (100.0)	130 (100.0)	83 (100.0)	117 (100.0)	387 (100.0)	1110 (100.0)

© 2020 Gaiha SM et al. *JAMA Network Open.*

eTable 4. Percentage of participants above and below the legal age purchasing e-cigarettes from locations before and after COVID-19

Location	Disposables						Pod-based e-cigarettes						Other e-cigarettes					
	Total sample		Under <21		Above 21		Total sample		Underage (<21)		Above 21		Total sample		Underage (<21)		Above 21	
	Before COVID (%)	After COVID (%)	Before COVID (%)	After COVID (%)	Before COVID (%)	After COVID (%)	Before COVID (%)	After COVID (%)	Before COVID (%)	After COVID (%)	Before COVID (%)	After COVID (%)	Before COVID (%)	After COVID (%)	Before COVID (%)	After COVID (%)	Before COVID (%)	After COVID (%)
**N**	**706**	**632**	**393**	**350**	**313**	**282**	**900**	**799**	**508**	**449**	**392**	**350**	**629**	**560**	**348**	**308**	**281**	**252**
Smoke shop or vape shop	26.8	17.9	26.5	18.3	27.2	17.4	27.0	18.0	27.4	18.7	26.5	17.1	32.4	23.4	37.1	27.3	26.7	18.6
Gas station	24.4	22.8	27.7	25.7	20.1	19.1	23.9	20.6	27.2	23.6	19.6	16.9	18.6	17.9	21.5	19.5	14.9	15.9
Online	16.1	33.1	13.7	30.3	19.2	36.5	18.6	36.5	17.5	35.4	19.9	38.0	17.8	35.5	15.8	33.1	20.3	38.5
Convenience store (like 7/11 or local mart)	11.2	9.5	11.5	11.4	10.9	7.1	11.6	8.3	11.4	9.3	11.7	6.9	9.7	7.7	8.6	8.1	11.0	7.1
Drugstore (like Rite Aid)	7.8	5.4	7.4	3.1	8.3	8.2	8.0	6.0	7.5	4.0	8.7	8.6	6.7	4.8	5.2	2.6	8.5	7.5
Liquor store	7.2	6.2	6.4	6.6	8.3	5.7	5.4	5.6	3.9	4.9	7.4	6.6	6.0	4.1	5.7	3.6	6.4	4.8
Medical marijuana dispensary	2.7	2.1	3.3	2.0	1.9	2.1	2.4	2.5	1.8	1.8	3.3	3.4	3.8	2.3	2.0	2.3	6.0	2.4
Supermarket (like Safeway)	2.3	1.4	2.5	1.1	1.9	1.7	0.9	1.2	0.8	1.1	1.0	1.4	2.2	2.1	2.6	2.6	1.8	1.6
Big retail store (like Walmart)	1.6	1.7	1.0	1.4	2.2	2.1	2.2	1.1	2.6	1.1	1.8	1.1	2.7	2.1	1.4	1.0	4.3	3.6

© 2020 Gaiha SM et al. *JAMA Network Open.*
